# Citation analysis of puerperal and menstrual psychosis

**DOI:** 10.1007/s00737-016-0673-x

**Published:** 2016-10-06

**Authors:** Ian Brockington

**Affiliations:** University of Birmingham, Edgbaston, Birmingham, B15 2TT UK

**Keywords:** Scholarship, Arrogance, Recognition, Neglect, International acclaim

## Abstract

A citation analysis of more than 2500 works on the childbearing and menstrual psychoses has shown that the average number of citations is 1 % of the literature or less; Anglo-Saxon authors have the lowest standards of scholarship. Many excellent works have received few of no citations. Attention is drawn to citation fraud and the pernicious effect of superficial “reviews.”

## Introduction

The psychoses of childbearing, first recognized at the dawn of medical science, and the menstrual psychoses, also described nearly 200 years ago, provide an opportunity to study the growth and spread of knowledge. That is the purpose of this citation analysis.

The assumption is that authors (no doubt, with exceptions), in order to underpin their statements and exhibit their erudition, will cite works they have obtained, unless they consider them trivial. The corollary—that they have carefully read what they have cited—is by no means true: obtaining and citing a publication certainly does not mean that it has been studied.

The citations of 2205 publications on childbearing psychosis and 357 on menstrual psychosis were indexed. This would have been a daunting task, except that about one third of the authors cited no publications, and the average number was in single figures.

## Indices

The number of citations made, and received, for each article, book, or thesis, was used to derive the following indices:

1. A measure of scholarship, based on mean citations made by various language groups (with the number of publications in that language as the denominator).
2. An index of linguistic isolation or arrogance, based on the proportion of citations in the author’s language (with the number of publications that have at least one citation as the denominator).
3. An index of recognition or neglect, based on the number of citations received.
4. For individual works, an index of international acclaim, based on the number of citations by other language groups.

## Citation fraud

In this literature, there are two remarkable examples of cheating. The bibliography of a French thesis (Lallier 1892) listed over 280 references—approximately 44 % of those published. Born in 1865, the author was only 27 years of age and would have had but a few years to collect this literature. There are various points to make as follows:

- The number of references, and proportion of works published, was much higher than any other nineteenth century writer.
- The French language was the fourth by frequency (52 references) after German (75), British (68), and American (59). It is unusual for French writers (or indeed authors from other nations) to cite foreign works more often than their own.
- Few Frenchmen cited American works, and only one cited as many as three. Lallier cited 59 (out of 99 published by 1891), which is almost as many as one of the best of the American reviews (Boyd 1942), who cited 63, writing 50 years later; Lallier cited some I was unable to find after searching for over 30 years, with the help of inter-library loans and friendly American libraries. Indeed, his list included two American papers (Kunst 1878; Lee 1881) that are said not to exist in the USA.

In the text, he mentioned only two American works (Macdonald 1831; Hirst 1888).

A few years later, another thesis (Castin 1899) went further: his bibliography included over 400 works, including 104 American articles (much more than has ever been cited by an American); in the text, he mentioned 46 articles including two in American.

There can be no doubt that these authors copied titles from one of the American inventories that had recently appeared—probably the Index Catalogue of the Surgeon General (which started in 1885). A bibliography is not a list of all works known to exist—it is testimony to the scholastic activities of the writer. Lallier’s and Castin’s bibliographies are a species of academic fraud. Their significance here is that they underline the fact that citation lists exaggerate the reading that has been done.

## Scholarship

In the literature on puerperal psychosis, the median number of citations was only 6, mean 9 (0.3 % of the literature). Only eleven authors cited more than 10 % of works published up to 1 year before their own (Marcé 1858; Rocher 1877; Lallier 1892; Weebers 1893; Senlecq 1896; Anton 1910; Meyer 1911; Zilboorg 1928; Duval 1934; Vayssière. 1949; Dazzi 1957), with Marcé at the top of the list (19 %); since Dazzi’s thesis, no publication has cited more than 10 % of the literature.

The corresponding figure for menstrual psychosis is median 2, mean 4.7 (1 % of the literature); ten authors cited at least 20 % of the prior literature (Berthier 1874; Powers 1883; Icard 1890; Schwob 1893; Kowalewski 1894; Epstein 1897; v. Krafft-Ebing 1902; Häffner 1912; Jolly 1914; Splett 1996) with Berthier (21/41 = 56 %, of which 17 were French) and v. Krafft-Ebing (54/11 = 48 %, of which 28 were German) at the top of the list.

### Language groups

Table 1 compares language groups (with more than 30 citations) in the order of the mean citations made. These results conceal large differences:

**Table 1:** Citations by language group

Language group	Psychoses of childbearing			Menstrual psychosis		
	Mean citations		Proportion in own language (%)	Mean citations		Proportion in own language (%)
	Made	Received		Made	Received	
Italian	15.5	2.4	16	5.8	0.9	2
Dutch	14.5	4.3	7	6.0	1.5	3
Nordic	9.3	8.7	4	2.2	6.6	10
French	8.1	6.7	47	2.7	3.9	73
German	7.6	6.6	32	5.7	2.7	87
Japanese	6.5	1.8	19	6.6	4.0	31
British Commonwealth	5.2	7.3	67	2.4	2.3	57
American	4.4	5.6	45	3.6	3.0	80

- In the literature on childbearing, French theses made the most citations (27) and other French works the least (3.7)
- There was a sharp drop in German citations received after 1914.

They show, however:

- A low level of scholarship: even the highest rates are low (27 cited works is a small number for a thesis); medians of 6 and 2, with means of 0.3 and 1 % of the literature are extremely low.
- This is especially true of the British Commonwealth and USA.
- The Anglo-Saxon nations had high rates of citations in their own language. For menstrual psychosis, this was also true of French and German authors. Italian and Dutch authors were more scholarly and less arrogant.

## International acclaim

On childbearing psychoses, 18 publications received at least 60 citations from language groups other than their own; in this computation, American and British Commonwealth publications have been considered as two separate groups, which accounts for 11/18 being Anglo-Saxon works. Only three had more than 100 citations from other language groups—Kendell (Kendell et al. 1987) with 112, Esquirol (Esquirol 1838) with 144—available in the English translation—and Marcé (Marcé 1858) with 161. The international acclaim received by two British authors is interesting: a Scottish study of infective delirium (Campbell Clark 1887) received 62 foreign and only one British citation, and the original description of eclamptic psychosis *sine* seizures (Donkin 1863) had 19 foreign and no British citations.

On menstrual psychosis, v. Krafft-Ebing’s work is the most cited, with 87 in all; he is the only author to receive more than 20 citations from non-German authors and has even been cited twice by American authors, but never before by an Englishman. It would be justifiable to regard citing his book as a marker for scholarship; no one who has not read it can claim knowledge of this subject. But it has been forgotten by some German authors (Schneider 1925; Hirschmann-Wertheimer 1927; Aschner 1931; Goldschmidt 1935; Martinius 1992), and, since 1925, has been cited only twelve times (Tagawa 1934; Horwitz and Harris 1935; Shrijver-Herzberger 1935; Vencovský 1937; Staabs 1939; Burckhart 1941; Knaus 1949; Bleuler 1954; Yamashita et al. 1962; Yamashita 1993; Abe & Ohta 1995; Stein et al. 2003).

## Under-cited works

There is a long list of important publications, which have received few citations. They include a dozen pioneering or sedulous studies of organic psychoses, including the first descriptions of parturient (Kirkland 1774) and postpartum delirium (Barth 1828), postpartum stupor (Kelso 1840; Tott 1844) five major articles on eclamptic psychosis, and the Japanese description of hyperammonemic psychosis (Yamada et al. 1980). An excellent thesis on chorea psychosis (Breton 1893) has received one citation. As for major works on non-organic psychoses, the marvelous original description of puerperal mania (Osiander 1797) has received 15 citations. The two largest series (Visscher 1949; Widerøe 1903) received one and five citations. A superb Dutch thesis (Van Steenbergen-van der Noordaa 1941) received three citations and two massive case series (Bonse 1989; Daseking 1931), with description of all cases and long follow-ups, have never been cited. A unique investigation by serial uterine biopsies (Delay et al. 1948) has been cited 30 times, but only 15 times by French authors, among 149 that have published in that language since 1950; French psychiatrists came under the influence of psychoanalysis, and this promising line of research petered out. On menstrual psychosis, important German papers have been neglected (Mendel 1881; Wollenberg 1891; Ewald 1922, 1924) or cited only by a few German authors. Half of the 38 Japanese papers have never been cited by a non-Japanese author; they include Yamashita’s *Periodic psychosis of adolescence* (Yamashita 1993), which has been translated into English.

## “Reviews”

The purpose of reviews is to find out what is known and to lay a foundation for future research. Their value depends on the number of publications reviewed, the inclusion of those written in foreign languages, and the quality of analysis and study. This literature includes over 400 reviews on childbearing psychoses, and it is obvious that this opinion on their purpose is not shared, because the median number of cited works is nine. Only one review (Anton 1910) had more than 100 references; it covered 14 % of the existing publications.

To fulfill their function, reviews should augment earlier work. An incomplete review is worse than worthless because it buries primary observations under a rootless pseudo-literature. For example, a recent “review of postpartum psychosis” (Sit et al. 2006) cited 45 relevant works, all in the English language, none written before 1969—about 2 % of the literature and only 10 % of the English language publications in the 35 years covered; although important details were missed, it has already been cited many times, presumably in the belief that it is authoritative. In 1930, Sir Thomas Lewis wrote,

There is little or no attempt of editors collectively to stem the tide of pseudo-scientific publication. By its mass it conceals work that has value; by its quality it undermines the general standard of accuracy in observation and thought.

The literature on the psychoses related to the female reproductive process is spread over at least two centuries and published in many languages. Editors should immediately reject “reviews” limited to recent publications in one language. Universities, when awarding doctorates, should insist on wide reading.

## Discussion

The aim of all scientific activity is the increase of knowledge. During the twentieth century, in which almost every area of medicine made astonishing and revolutionary progress, knowledge of the psychoses of childbearing and menstruation has stagnated.

Mothers have benefited from advances in obstetrics and infection control, which have greatly reduced the frequency of organic psychoses. The duration of non-organic psychoses has been reduced from several months to as many weeks by ECT and neuroleptic agents, and the development of in-patient mother and baby units has also been favorable. But there have been scarcely any advances in our knowledge of the cause of childbearing psychoses:

- As the organic psychoses diminished, it became clearer that the non-organic psychoses were more common in first-time mothers, although this is a small effect and applies only to those of early postpartum onset.
- With a shift in nosological boundaries, the link between non-organic postpartum psychosis and bipolar disorders was perceived, leading to treatment and prevention by lithium and other mood stabilizers. There is evidence from genetic studies, and the long-term studies (Brockington 2014), that puerperal and other triggers of bipolar/cycloid disorder are distinct.

Although, in an era of “biological psychiatry,” the childbearing psychoses are a fertile field for neuroendocrinological and other neuroscientific studies, there have been no replicated findings.

On the menstrual psychoses, it was known by 1902 that there was an association between psychosis and the menses, and cases had already been observed during amenorrhoea, after childbirth, before and at the menarche, and in men; but this knowledge has been lost—many psychiatrists do not recognize the existence of these disorders. Before 1850, French flare had spotted the link, and before 1930, German thoroughness provided a solid clinical base for further studies. Surveys were the next stage, and building up registers for genetic and epidemiological study. After 1960, treatment trials and neuroendocrinological studies became possible. This has not happened. In 2015, we are still at baseline, approaching the problem anew. As in the early nineteenth century, isolated authors are publishing sporadic cases, without having gained anything from the lessons learned about clinical methodology 100 years ago.

A potent reason for stagnation is this neglect of the literature. Even the few publications that have appeared have not been studied. Knowledge is gained by field studies and by studying completed research, which are non-competitive and complementary activities. But the culture of academic medicine values only research activity—obtaining funds, field-work, publication in “high impact journals” citation, and foreign invitations.

It is absurd to plan, fund, or conduct research without locating the growing point and ridiculous to publish work (in the expectation that other scientists will respect and study it) without reciprocation. This citation analysis has exposed the depth and degree of this abysmal and universal neglect. Neglect of the literature has clinical as well as scientific consequences: there are striking examples of poor care because what has long ago been discovered is no longer known (Brockington 2016).
